# A Case of Refractory Tracheocutaneous Fistula Successfully Treated With the Combination of Auricular Cartilage Grafting and Sternocleidomastoid Muscle Flap

**DOI:** 10.7759/cureus.65345

**Published:** 2024-07-25

**Authors:** Kyoko Baba, Akinari Yoshizawa

**Affiliations:** 1 Department of Plastic and Aesthetic Surgery, School of Medicine, Kitasato University, Sagamihara, JPN; 2 Department of Plastic Surgery, Kitasato University Medical Center, Kitamoto Saitama, JPN

**Keywords:** sternocleidomastoid muscle flap, tracheal cartilage defect, auricular cartilage grafting, tracheocutaneous fistula closure, tracheocutaneous fistula

## Abstract

The tracheotomy site usually closes spontaneously after decannulation, but in rare cases, it develops into tracheocutaneous fistula. We experienced a case of tracheocutaneous fistula that was successfully treated with the combination of auricular cartilage grafting and sternocleidomastoid muscle flap. In this case, we performed the closure of tracheocutaneous fistula with a view to filling the tissue defect with soft tissue to prevent recurrence. The surgical procedure performed in this case was unique, which to our knowledge, has not been described previously. Herein, we report some findings obtained, together with a literature review.

The patient was a 73-year-old male. Starting five months after tracheotomy, the closure of a tracheocutaneous fistula was attempted twice at an otolaryngology clinic, which resulted in recurrence. The patient visited our department with the desire to close the tracheocutaneous fistula. At the initial examination, we found a cutaneous fistula with a diameter of approximately 2 mm on the cranial side of the sternal notch and thinning of the surrounding tissue. Preoperative computed tomography (CT) showed a tracheal defect with a size of approximately 10 mm on the caudal side of the sternal notch. The surgery was performed under general anesthesia 10 months after tracheotomy. The platysma muscle was attached to elevate the skin flap, and the scarring at the cutaneous fistula opening was removed. The cartilage defect was 10×12 mm in size. A piece of cartilage was harvested from the posterior surface of the auricle (navicular fossa) and grafted to the tracheal opening. A part of the left sternocleidomastoid muscle body of the sternal head was dissected from the mandibular side using the sternal attachment site as a stalk and elevated. The muscle flap was rotated, with its tip folded back, doubled over, and fixed on top of the auricular cartilage graft. The platysma muscles were sutured together during which the skin flap suture line was shifted so that the suture line would not coincide with the tracheal fistula site. The course was favorable, with no recurrence for three years.

In the closure of a tracheocutaneous fistula, two sides need to be considered: the trachea and the skin. The tracheal defect in the present case was larger than 10 mm in size and thus auricular cartilage grafting was performed. In addition, we filled the tissue defect with the soft tissue of a sternocleidomastoid muscle flap, which was a unique step. The combined use of auricular cartilage grafting and sternocleidomastoid muscle flap was effective for the closure of a refractory tracheocutaneous fistula.

## Introduction

The tracheotomy site usually closes spontaneously after decannulation, but in rare cases, it develops into a tracheocutaneous fistula, such as in patients with long-term tracheotomy. Since tracheocutaneous fistula reduces the quality of life (QOL) of patients, patients normally undergo fistula closures. However, simple closure with fistulectomy often results in the recurrence of tracheocutaneous fistula due to wound disruption or mediastinal emphysema due to incomplete closure of the tracheostomy [1-3]. A previous report showed that the overall incidence of tracheocutaneous fistula was 3.3-29% and 70% occurred in patients with over 16 weeks of tracheotomy [4]. If there is difficulty in closing a tracheocutaneous fistula, the patient may be referred to the plastic surgery department for the purpose of fistula closure. A variety of surgical procedures have been reported for the closure of tracheocutaneous fistula, including free cartilage grafting, local skin valvuloplasty, and free musculocutaneous flap operation [5-8]. In order to close a tracheocutaneous fistula, these reports considered the reconstruction of two sides: the trachea and skin (surface layer). In the present case, we not only performed the conventional two-sided reconstruction but also added the step of filling the tissue defect with soft tissue to minimize the chance of recurrence, presenting a unique surgical procedure. Following two unsuccessful attempts of tracheocutaneous fistula closure, we were able to successfully close the tracheocutaneous fistula using a combination of auricular cartilage grafting and sternocleidomastoid muscle flap. To our knowledge, there have been no reports of the surgical procedure performed in this case. Herein, we report some findings obtained.

## Case presentation

A 73-year-old male presented with a chief complaint of the desire to close the tracheocutaneous fistula so that he could take bath in the bathtub. The patient had a history of bilateral chronic suppurative otitis media with cholesteatoma, post-operation gallstone cholecystitis, post-endoscopic retrograde cholangiopancreatography (ERCP) severe acute pancreatitis, hyperlipidemia, and benign prostatic hypertrophy. The patient was diagnosed with post-ERCP acute pancreatitis and acute heart failure. He was intubated and a tracheotomy was performed for systemic management. The patient underwent decannulation after 8 weeks. At an otolaryngology clinic, fistulectomy and closure of the tracheotomy hole with simple sutures were attempted twice, both of which resulted in the recurrence of the fistula. The patient was referred to our department for the closure of the refractory tracheocutaneous fistula.

**Figure 1:**
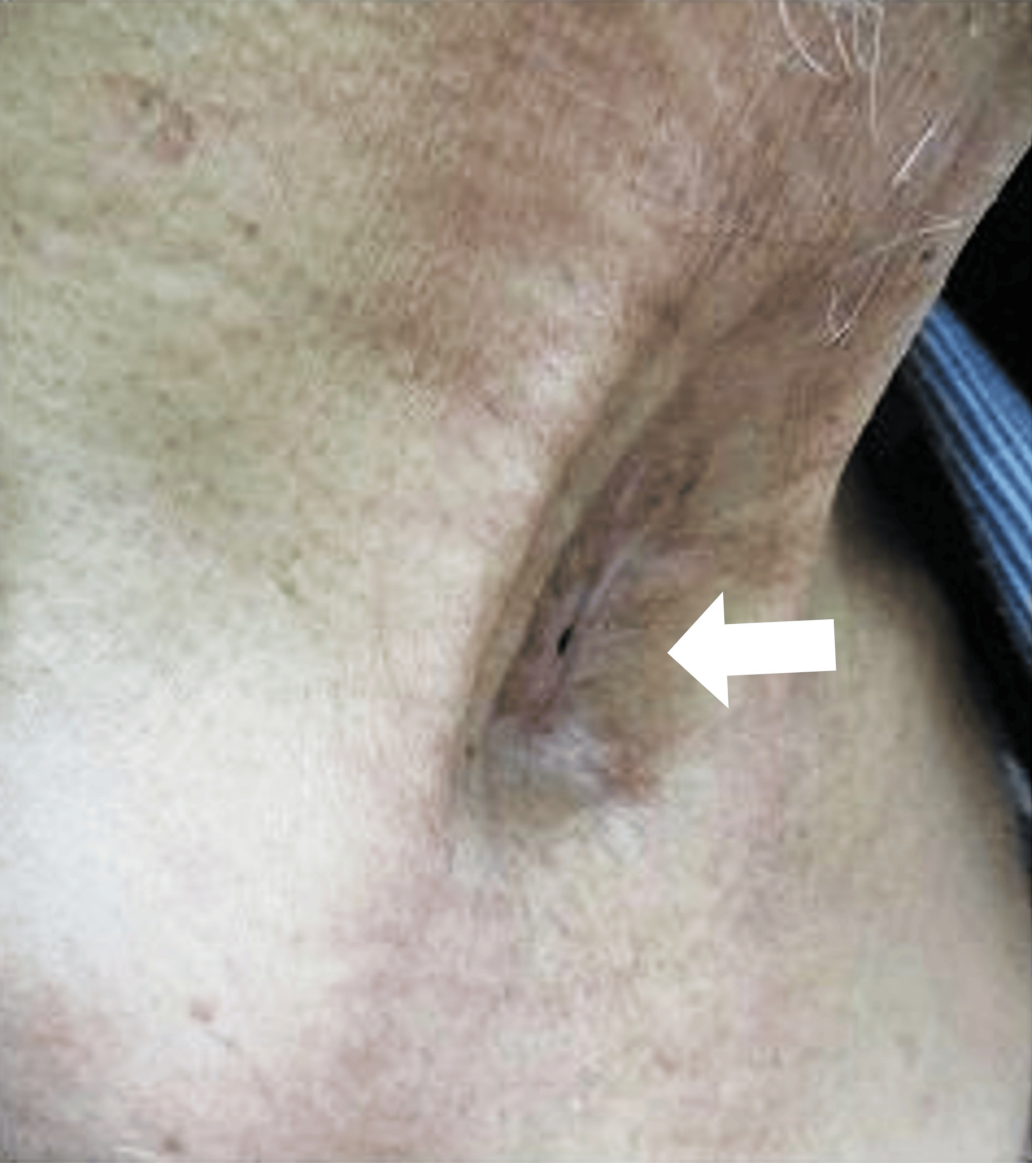
Findings at the initial examination A tracheocutaneous fistula opening with a diameter of approximately 2 mm was observed, as indicated by the arrow.

In the initial examination of the patient at our department, we observed a cutaneous fistula with a diameter of approximately 2 mm on the cranial side of the sternal notch and thinning of the surrounding tissue (Figure 1).

**Figure 2:**
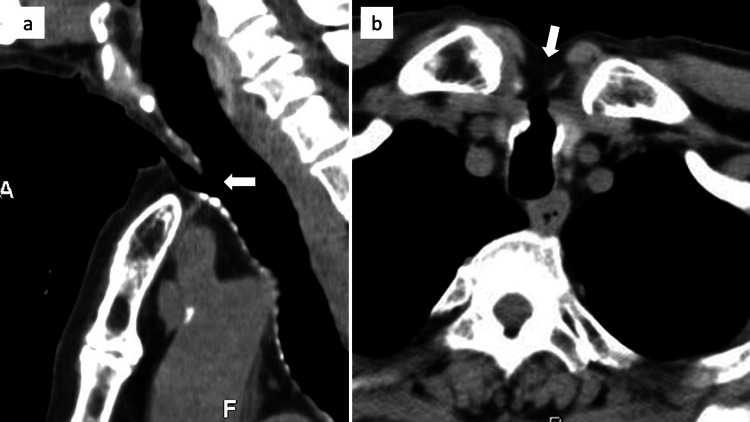
Preoperative computed tomography (CT) findings: (a) sagittal view; (b) axial view. A tracheal defect with a size of approximately 10 mm was observed caudal to the sternal notch, as indicated by the arrow.

Preoperative computed tomography (CT) showed that the tracheal defect with a size of approximately 10 mm was located slightly caudal to the sternal notch (Figure 2).

We planned to perform auricular cartilage grafting and closure of the tracheocutaneous fistula using a sternocleidomastoid muscle flap.

**Figure 3:**
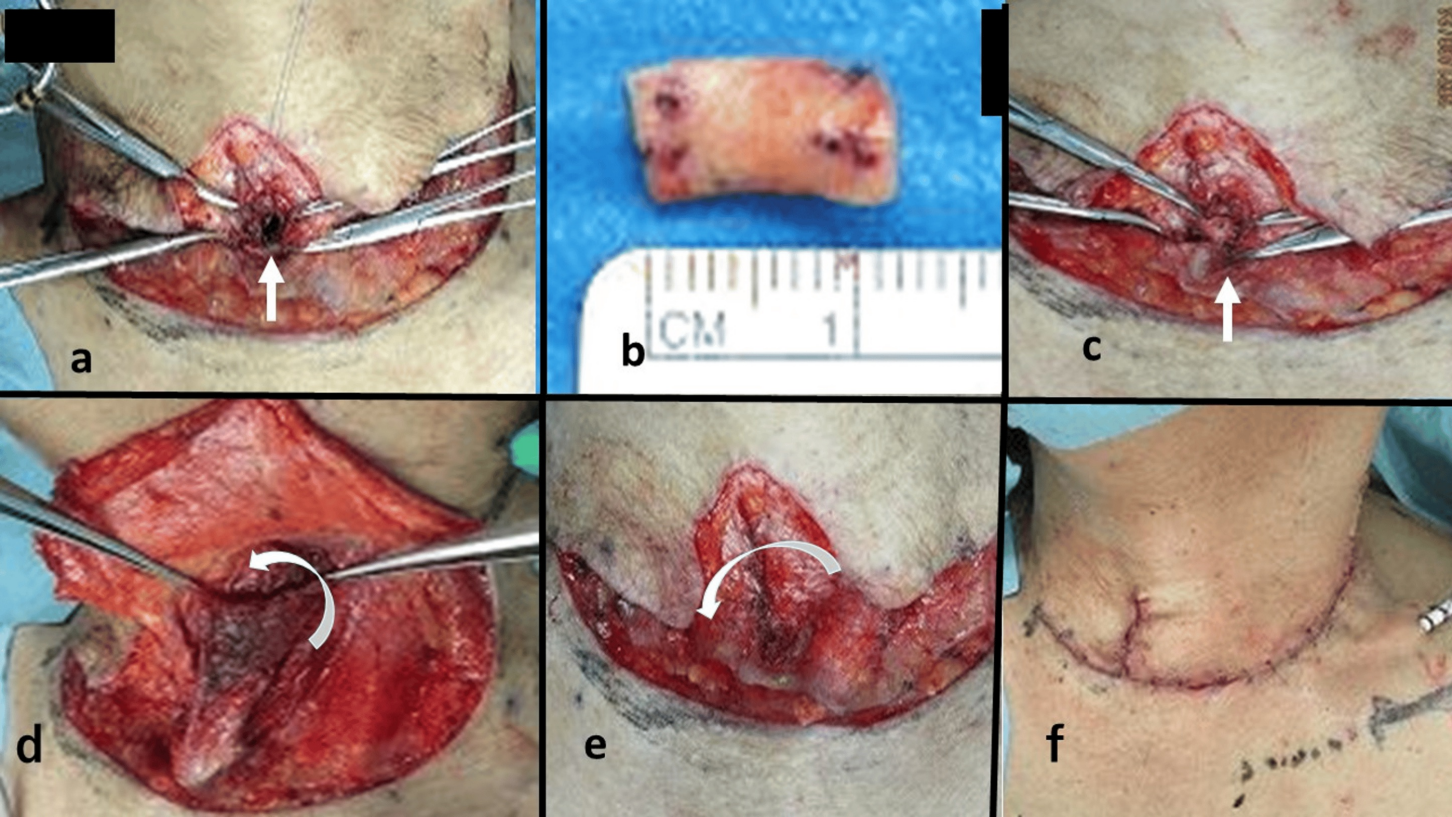
Surgical findings a. A tracheal cartilage defect with a size of 10×12 mm was observed, as indicated by the arrow. b. A piece of auricular cartilage with a size of 12 mm was harvested. c. A piece of auricular cartilage that had been processed according to the tracheal cartilage defect, as indicated by the arrow, was grafted. d. A part of the left sternocleidomastoid muscle body of the sternal head was dissected from the mandibular side using the sternal attachment site as a pedicle and elevated. As shown by the arrow, the muscle flap was rotated and moved onto the grafted cartilage at the tracheal defect. e. After the sternocleidomastoid muscle flap was fixed, the platysma muscles were sutured together. When suturing, the skin flap suture line was shifted to the right, as shown by the arrow, so that the suture line would not coincide with the tracheal fistula site. f. The condition at the end of the surgery is shown. A suction drain was placed.

The surgery was performed under general anesthesia 10 months after tracheotomy. During surgery, the patient was placed in an extended cervical position to expose the tracheal defect under the sternum. With a U-shaped incision, the platysma muscle was attached, and the skin flap was elevated. The thinned, scarred skin surrounding the cutaneous fistula opening was removed to the minimum extent necessary. The tracheal cartilage at the tracheal fistula opening was calcified with the cartilage flap being elevated, which was then removed. As a result, the tracheal cartilage defect was 10×12 mm in size (Figure 3a).

**Figure 4:**
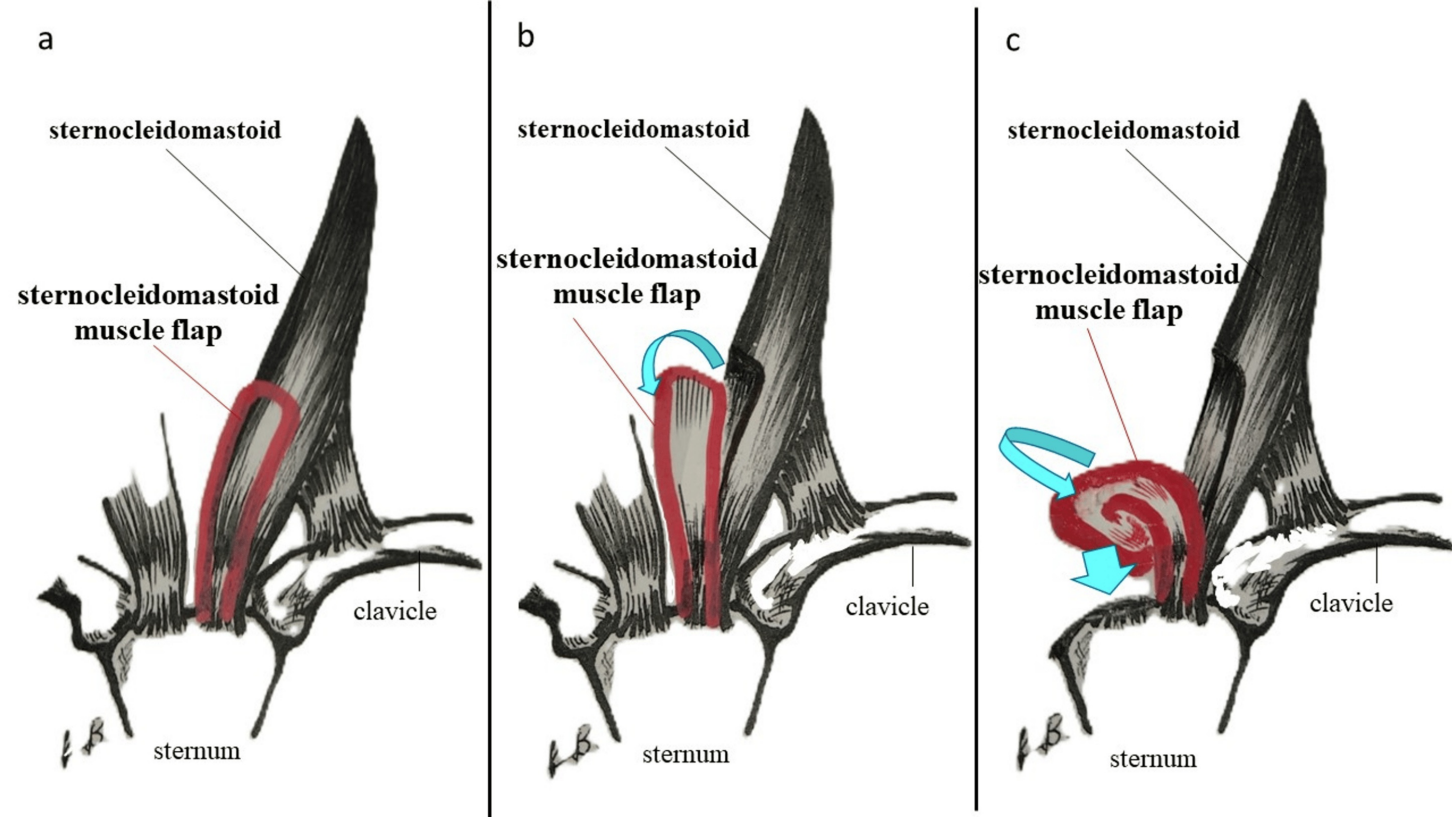
Schematic diagram of the sternocleidomastoid muscle flap a. A part of the left sternocleidomastoid muscle body of the sternal head was dissected from the mandibular side using the sternal attachment site as a stalk and elevated. b. The muscle flap was rotated and moved, as indicated by the arrow. c. The tip of the muscle flap was folded back and fixed on top of the auricular cartilage graft, as indicated by the arrow.

A piece of cartilage was harvested from the posterior surface of the left auricle (navicular fossa) (Figure 3b), processed according to the defect, and grafted to the tracheal opening (Figure 3c). Because the tracheal defect was located slightly to the left, we decided to use the left sternocleidomastoid muscle. The size of the muscle flap was determined by measuring the distance from the sternal attachment site of the left sternocleidomastoid muscle, which formed the base of the muscle flap, to the tracheal defect. A part of the left sternocleidomastoid muscle body of the sternal head was dissected from the mandibular side using the sternal attachment site as a stalk and elevated (Figure 3d). The muscle flap was rotated and moved to the tracheal defect, followed by folding the tip of the muscle flap back and fixing it on top of the auricular cartilage graft (Figure 4a-4c).

**Figure 5:**
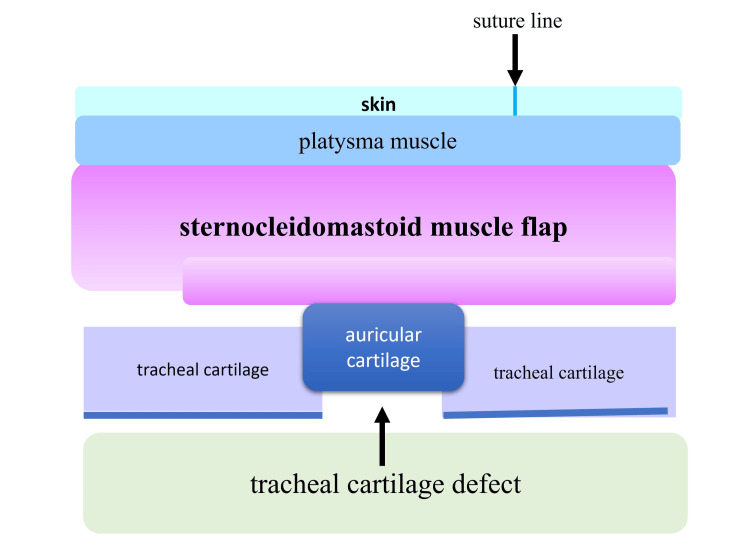
Schematic diagram of the postoperative cross-section By placing a double-muscle flap on top of the cartilage graft, we planned to fill the tissue defect, providing reinforcement to prevent the recurrence of the tracheal fistula. Additionally, the platysma muscles and skin were sutured, avoiding the fistula site immediately above.

By placing a double-muscle flap on top of the cartilage graft, we planned to fill the tissue defect, providing reinforcement to prevent the recurrence of the tracheal fistula. Furthermore, the platysma muscles were sutured together (Figure 3e), during which, the skin flap suture line was shifted slightly to the right so that the suture line would not coincide with the tracheal fistula site (Figure 3f). The schematic diagram of the postoperative cross-section is shown in Figure 5.

The surgical time was two hours and 27 minutes.

**Figure 6:**
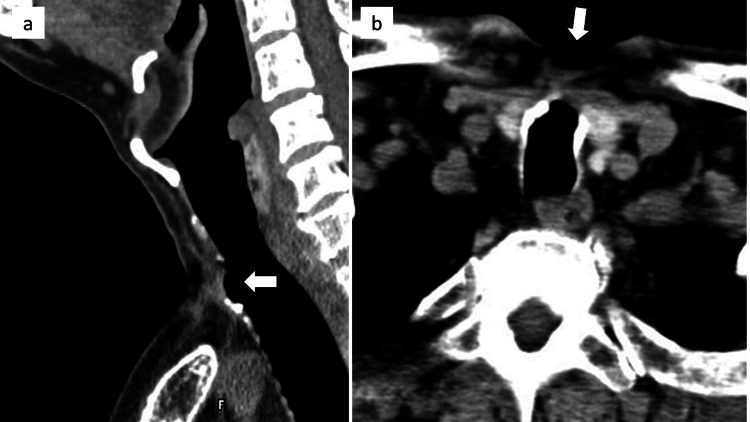
Postoperative computed tomography (CT): (a) sagittal view; (b) axial view. The tracheal cartilage defect was covered, and the amount of soft tissue reinforced by the sternocleidomastoid muscle was maintained.

The postoperative course was uneventful, with no postoperative complications. CT performed six months after surgery showed coverage of the tracheal cartilage defect, and the amount of soft tissue reinforced by the sternocleidomastoid muscle was maintained (Figure 6).

**Figure 7:**
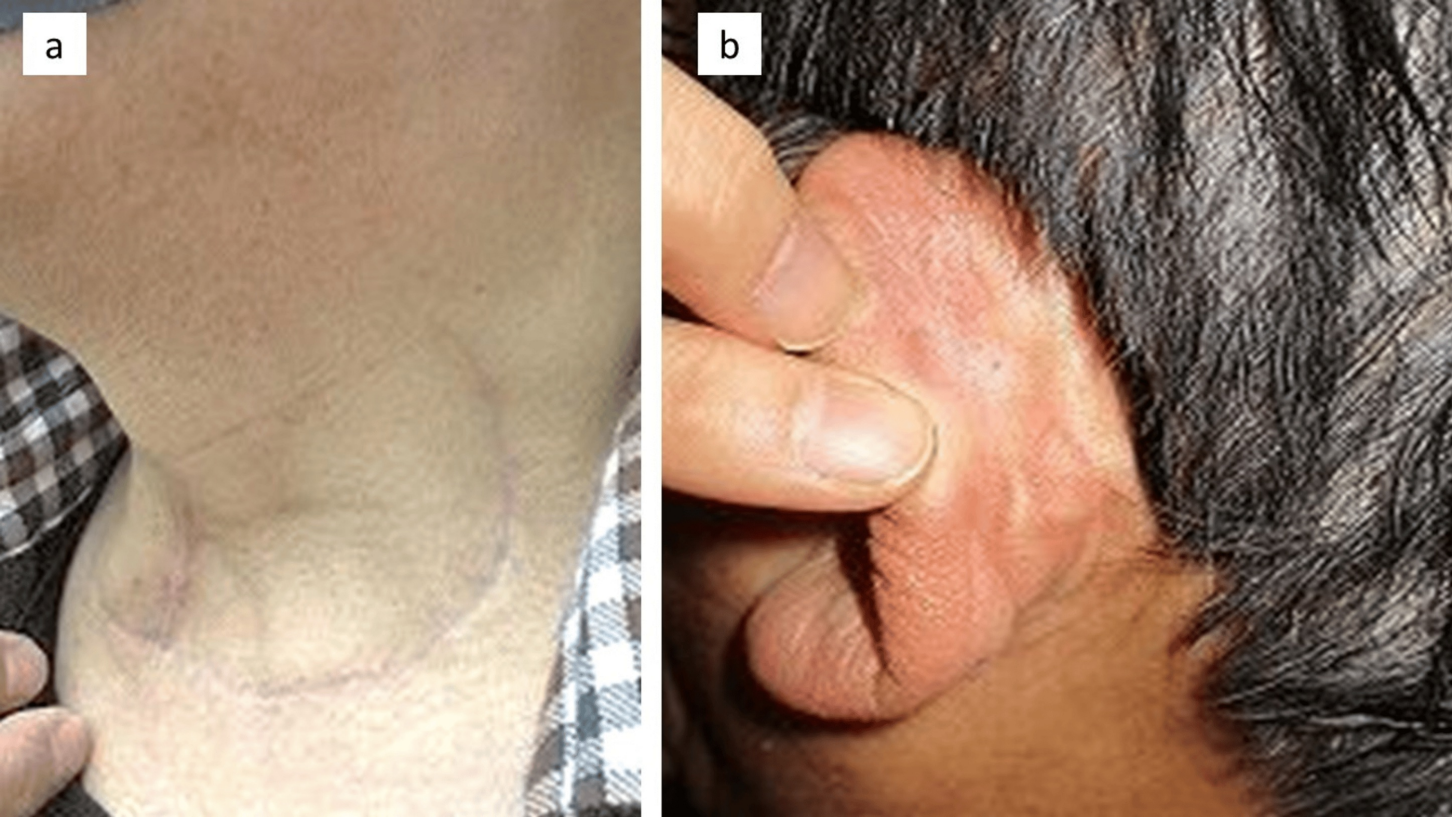
Postoperative findings There was no postoperative recurrence of tracheocutaneous fistula and no problem with scarring. a. We did not observe atrophy of the left sternocleidomastoid muscle. b. Finding of donor site: post-auricular site.

Three years have passed since the surgery, with no recurrence of tracheocutaneous fistula. We achieved a cosmetically satisfactory outcome without any problems of postoperative scarring or atrophy of the left sternocleidomastoid muscle (Figure 7).

## Discussion

The causes of tracheocutaneous fistula include prolonged tracheotomy, adhesion, and infiltration of thyroid cancer, post-radiation therapy, and circumferential suture of the trachea [9]. The basic treatment for tracheocutaneous fistula is surgical closure [10]. In general, simple closure for the primary fistula carries the risk of mediastinal emphysema and wound disruption. Therefore, the closure of two sides, the trachea and skin (body surface), is recommended [10].

Based on previous reports, tissues that can be used for the closure of the trachea include cartilage [11], tensor fasciae latae [12], palatal mucosa [13], local turnover skin flap [7,10,14], and platysma muscle flap [6]. For the closure of the skin (body surface), the use of a local skin flap [13], musculocutaneous flap [15], and free skin flap [8] has been reported.

According to the treatment algorithm reported by Kao et al. [15], cartilage grafting was not necessarily required in cases with a small tracheal cartilage defect. In addition, previous reports have shown that, for a small tracheal cartilage defect, the skin can be used to close the trachea with a hinge flap, and the skin can be closed with a local skin flap [7,10,14]. On the other hand, cartilage grafting is recommended if the tracheal defect is 10 mm or larger in size. The present case was a re-recurrent case with no shrinkage of the fistula for over three months and a tracheal defect with a size of over 10 mm, indicating the necessity of closure with cartilage grafting.

The uniqueness of our surgical procedure lies in that we not only performed the conventional two-sided reconstruction but also added the step of filling the tissue defect with soft tissue to minimize the chance of recurrence. Prior to performing the surgery, we determined the surgical procedure in consideration of not only the size of the cartilage defect but also the following aspects: (1) a re-recurrent case, (2) a scarce amount of soft tissue, and (3) possible adhesion and poor vascularity in the soft tissue around the tracheal fistula. Furthermore, (4) the tracheal defect in this case was located below the sternal notch, and because local flaps using the skin, such as hinge flaps, had limited movement, there was a possibility that a skin flap would not reach the tracheal defect. To address these issues, in addition to auricular cartilage grafting, we planned a surgical procedure of cartilage grafting that used a nearby sternocleidomastoid muscle flap to fill the tissue defect with soft tissue. Moreover, to address issues (2) and (3) mentioned above, we planned to fix the soft tissue to the upper layer of the grafted cartilage in this re-recurrent case. We expected that this would improve the survival of free tissue, that is, the grafted cartilage with no vascularity, and prevent infection.

We selected a nearby sternocleidomastoid muscle with good blood flow as the soft tissue for filling the tissue defect. The sternocleidomastoid muscle flap is sometimes used in the reconstruction of the head and neck region [16]. The sternocleidomastoid muscle originates from the sternum (sternal head) and clavicle (clavicular head), as well as ends at the mastoid process of the temporal bone and occipital bone. Its nutrient vessels are the occipital and external carotid arteries on the cranial side, as well as the superior thyroid artery on the caudal side. Its dominant nerves are accessory and cervical nerves (C2, C3), which enter the muscle body cranially from the bifurcation of the common carotid artery [17]. Considering these anatomical characteristics of the sternocleidomastoid muscle, we used a necessary and sufficient amount of the muscle caudal to the bifurcation of the common carotid artery. This allowed us to preserve blood flow to the muscle and avoid damage to the dominant nerves.

The surgical procedure used in this case involved relatively simple operations with minimal surgical invasiveness and was effective in treating a re-recurrent case of refractory tracheocutaneous fistula.

## Conclusions

We successfully treated a case of refractory tracheocutaneous fistula using a combination of auricular cartilage grafting and sternocleidomastoid muscle flap. The tracheal cartilage defect in this case was larger than 10 mm in size, requiring free auricular cartilage grafting. In addition to the conventional two-sided reconstruction of the skin and trachea, our unique method fixed soft tissue to the site of tracheocutaneous fistula closure to prevent recurrence. For this, we used a part of the sternocleidomastoid muscle body caudal to the bifurcation of the common carotid artery. This simple, convenient, and minimally invasive surgical procedure was effective as a method for closing refractory cutaneous tracheal fistula in a re-recurrent case.
